# Interaction between lead and noradrenergic genotypes affects neurocognitive functions in attention-deficit/hyperactivity disorder: a case control study

**DOI:** 10.1186/s12888-020-02799-3

**Published:** 2020-08-06

**Authors:** Jae-Won Choi, A-Hyun Jung, Sojeong Nam, Kyoung Min Kim, Jun Won Kim, Soo Yeon Kim, Bung-Nyun Kim, Jae-Won Kim

**Affiliations:** 1 Department of Psychiatry, Gyeongsang National University Hospital, Jinju, Republic of Korea; 2 Suyeong-gu Mental Health Service Center, Busan, Republic of Korea; 3 Department of Rehabilitation and Counselor Education, University of Iowa, Iowa City, IA USA; 4 Department of Psychiatry, Dankook University College of Medicine, Cheonan, Republic of Korea; 5 Department of Psychiatry, Catholic University of Daegu School of Medicine, Daegu, Republic of Korea; 6 Department of Psychiatry, Pusan National University Hospital, Busan, Republic of Korea; 7 Division of Child and Adolescent Psychiatry, Department of Psychiatry, Seoul National University College of Medicine, 101 Daehak-No, Chongno-Gu, Seoul, Republic of Korea

**Keywords:** Lead, Neurocognitive performance, ADHD, Dopamine, Noradrenaline

## Abstract

**Background:**

Lead is known to be associated with attention-deficit/hyperactivity disorder (ADHD) even at low concentrations. We aimed to evaluate neurocognitive functions associated with lead in the blood and the interactions between lead and dopaminergic or noradrenergic pathway-related genotypes in youths with ADHD.

**Methods:**

A total of 259 youths with ADHD and 96 healthy controls (aged 5–18 years) enrolled in this study. The Korean Kiddie Schedule for Affective Disorders and Schizophrenia–Present and Lifetime version was conducted for psychiatric diagnostic evaluation. Blood lead levels were measured, and their interaction with dopaminergic or noradrenergic genotypes for ADHD; namely, the dopamine transporter (*DAT1*), dopamine receptor D4 (*DRD4*), and alpha-2A-adrenergic receptor (*ADRA2A*) genotypes were investigated. All participants were assessed using the ADHD Rating Scale-IV (ADHD-RS). Participants also completed the continuous performance test (CPT) and Stroop Color-Word Test (SCWT). Analysis of covariance was used for comparison of blood lead levels between ADHD and control groups. A multivariable linear regression model was used to evaluate the associations of blood lead levels with the results of ADHD-RS, CPT, and SCWT; adjusted for intelligence quotient (IQ), age, and sex. A path analysis model was used to identify the mediating effects of neurocognitive functions on the effects of blood lead on ADHD symptoms. To evaluate the effect of the interaction between blood lead and genes on neuropsychological functions, hierarchical regression analyses were performed.

**Results:**

There was a significant difference in blood lead levels between the ADHD and control groups (1.4 ± 0.5 vs. 1.3 ± 0.5 μg/dL, *p* = .005). Blood lead levels showed a positive correlation with scores on omission errors(r = .158, *p* = .003) and response time variability (r = .136, *p* = .010) of CPT. In the multivariable linear regression model, blood lead levels were associated with omission errors (B = 3.748, *p* = .045). Regarding the effects of lead on ADHD symptoms, hyperactivity-impulsivity was mediated by omission errors. An interaction effect was detected between *ADRA2A* DraI genotype and lead levels on omission errors (B = 5.066, *p* = .041).

**Conclusions:**

Our results indicate that neurocognitive functions at least partly mediate the association between blood lead levels and ADHD symptoms, and that neurocognitive functions are affected by the interaction between blood lead levels and noradrenergic genotype.

## Background

Attention-deficit/hyperactivity disorder (ADHD) occurs in 5% of children [1] and is characterized by symptoms of hyperactivity, impulsivity, and inattention leading to functional impairments in social activities, performance at school, and family relationships [2]. Despite the complex etiology of ADHD, there is a general consensus that ADHD is underpinned by environmental and genetic factors [3, 4]. Among psychiatric disorders, ADHD is considered a highly heritable disorder with an estimated genetic heritability of 0.77 [4]. Many candidate genes, including dopaminergic, serotonergic, and noradrenergic pathway-related genes, are reported to be associated with ADHD [5, 6]. However, no single gene has yet been identified as a risk factor for the development of ADHD, because of small effect sizes or insufficient sample sizes [7]. In a few genome-wide studies, the association between specific SNPs and ADHD have been replicated [6]. Notwithstanding the limited genetic studies, several genes of interest have been identified. Genes in the dopaminergic pathway, such as the dopamine transporter (*DAT1*) and dopamine D4 receptor (*DRD4*), are most commonly reported as having positive associations with ADHD [6]. Recent imaging studies have focused on these two genes [8]. Noradrenergic genes, such as the alpha-2A adrenergic receptor gene (*ADRA2A*), have been studied in the context of their relationship with symptoms and executive functions in ADHD [9, 10].

Genetic risk factors alone cannot explain the course and development of ADHD [3, 7]. Alongside genetic risk factors, environmental risk factors account for an estimated 10–40% of the variance of the disorder [11]. In previous studies, environmental risk factors such as lead, manganese, or polychlorinated biphenyls correlated with symptoms of inattention and hyperactivity [3, 5]. However, genetic and environmental risk factors do not independently influence symptomatology of, and vulnerability to ADHD. While environmental risk factors can affect the expression of genetic factors, genetic factors can also increase the likelihood of exposure and susceptibility to environmental factors [7]. In this context, gene–environment (G-E) interactions can provide explanations for differential sensitivities of ADHD development [12]. Although the effects of G-E interactions on ADHD have been investigated [13], a limited number of studies have supported this hypothesis, or identified the specific functions or symptoms affected by G-E interactions. Indeed, the majority of previous reports have not been replicated [14]. According to a recent hypothesis, abnormalities in fronto-subcortical pathways caused by G-E interactions may influence neuropsychological endophenotypes, which can be measured by neuropsychological tests such as the continuous performance test (CPT) and Stroop Color-Word Test (SCWT). These neuropsychological endophenotypes may be presented as ADHD symptoms [15].

Lead is a well-known neurotoxicant that can damage multiple organs [16] and interfere with neurocognitive development [17]. As the lead absorption rate is higher in children than in adults and can easily cross the blood-brain barrier [18], children are especially susceptible to lead toxicity. In addition, even at levels lower than 10 μg/dL, it has been reported that lead exposure is associated with reduced intelligence, impaired academic achievement, and executive functioning [19, 20]. An association of clinical diagnosis and symptoms of ADHD with low lead levels has been reported [14, 21]. One study suggested that blood lead level is more strongly associated with hyperactivity/impulsivity symptoms than inattention [21], but a recent meta-analysis indicated that both symptoms exhibit a similar relationship with lead exposure [22]. In studies on associations between lead exposure and genes, an interaction between lead exposure and the N-methyl-d-aspartate (NMDA) receptor gene was identified to affect memory [23, 24]. The *DAT1* gene is an ADHD candidate gene reported to be associated with response inhibition [25]. A study in rats reported a slight decrease in the expression of the DAT1 protein when exposed to lead, but there are few studies related to lead exposure [26]. In children lacking the *DRD4–7* polymorphism, impaired executive functions were linked to increased lead levels [27]. A recent study showed significant association between the *DAT1* VNTR and *DRD4* VNTR variants and ADHD in Koreans [28]. *ADRA2A* MspI and DraI polymorphisms were reported as predictors of treatment responses in ADHD with blood lead [15]. However, since the effects of lead exposure on ADHD symptoms were mediated more by poor performance on the stop task measuring response inhibition [21], it is necessary to examine which genes, of those demonstrating interactions with lead exposure, are correlated with executive functions that could mediate the effects of blood lead on symptoms of ADHD.

In this study, we aimed to evaluate which executive functions mediated the effects of blood lead levels on ADHD symptoms and the specific genes interacting with lead that were correlated with executive functions affected by blood lead levels. Based on previous studies, we hypothesized that lead, as an environmental risk factor, would interact with dopamine-related genes, such as *DAT1* and *DRD4*, and norepinephrine-related genes, such as *ADRA2A*, and that this interplay would affect specific executive functions, such as inattention, impulsivity, and processing speed.

## Methods

### Participants

Data from 355 participants comprising 259 patients with ADHD and 96 controls (aged 5–18 years) were pooled from two studies conducted with the same protocol between August 2010 and February 2015 at the child and adolescent psychiatry outpatient clinic of our hospital. In total, 90 patients with ADHD and 33 controls were initially recruited from the first study; after excluding two patients with ADHD and eight controls with missing genetic or environmental data, 88 patients with ADHD and 25 controls were assessed. In the second study, 191 patients with ADHD and 78 controls were initially recruited; after excluding 20 patients with ADHD and seven controls with missing genetic or environmental data, 171 patients with ADHD and 71 controls were assessed. A board-certified child and adolescent psychiatrist performed a psychiatric diagnostic evaluation, consisting of the Korean Kiddie Schedule for Affective Disorders and Schizophrenia–Present and Lifetime version (K-SADS-PL), for each participant in both groups. The validity and reliability of the Korean version of the K-SADS-PL have been ascertained [29]. Exclusion criteria for ADHD and control groups were: a hereditary genetic disorder; intelligence quotient (IQ) < 70; current or past history of brain trauma, organic brain disorder, seizure, or any other neurological disorder; schizophrenia or any other childhood-onset psychotic disorder; autism spectrum disorder, communication disorder, or learning disorder; major depressive disorder or bipolar disorder; obsessive compulsive disorder; Tourette’s syndrome or a chronic motor/vocal tic disorder. Detailed information about the study was provided to the parents and participants, and written informed consent was obtained from parents before enrolling any participants in the study. Both study protocols were approved by the Institutional Review Board of Seoul National University Hospital.

### Assessment of ADHD symptoms and neuropsychological functions

The Korean version of the ADHD Rating Scale-IV (ADHD-RS) was completed by the parents to assess the severity of ADHD symptoms [30]. The ADHD-RS consists of two subscales assessing symptoms related to inattention and hyperactivity/impulsivity. Attention and executive functions of participants were measured using the computerized CPT and SCWT. In the CPT, four variables were recorded: (1) omission errors interpreted as indicators of inattention, (2) commission errors interpreted as indicators of impulsivity, (3) mean reaction time interpreted as indicators of processing speed, and (4) standard deviations of the response times interpreted as indicators of variability or consistency of attention. The validity and reliability of the Korean version of the computerized CPT have been well-established [31]. The SCWT has been used to assess the ability to inhibit or ignore interference of irrelevant stimuli and was standardized in Korean [32]. To assess cognitive function, the Korean Educational Development Institute’s Wechsler Intelligence Scales for Children (KEDI-WISC) [33] was administered.

### Measurement of blood lead

From each child, 5 mL venous blood samples were collected in metal-free tubes for lead measurements. Matrix modifier reagent composed of Triton X-100 and ammonium hydrogen phosphate dibasic was used for dilution, and the atomic absorption spectrometer–graphite furnace (Analyst 800-Zeeman collection; PerkinElmer, Singapore) was used to assay lead concentrations. Using this method, the detection limit for lead was 0.058 μg/dL. The methods for the assay have been described previously [34].

### Genotyping

For genotyping of dopamine-related genes, a Genomic DNA Extraction Kit (Bioneer, Korea) was used to extract genomic DNA from blood lymphocytes. The 40-base pair VNTR polymorphism, located in the 3′-UTR of *DAT1* on chromosome 5p15.3, was genotyped using T7–5 Long (5′-TGT GGT GTA GGG AAC GGC CTG AG-3′) and T7-3a Long (5′-CTT CCT GGA GGT CAC GGC TCA AGG-3′). The genotypes of the *DAT1* variant were grouped according to the number of 10 repeats they carried as 10/10, with a 10, or without 10. The *DRD4* exon III VNTR polymorphism comprised 2–10 variable repeat units (1 unit = 48 base pairs) and was generated using the oligonucleotide primers (5′-ACC ACC GGC AGG ACC CTC ATG GCC TTG CGC TC-3′ and 5′-CTT CCT ACC CTG CCC GCT CAT GCT CTA CTG G-3′). The genotypes of the *DRD4* variant were grouped into 4/4, with a 4, and without 4. For genotyping of norepinephrine-related genes, G-DEX™ II Genomic DNA Extraction Kit (Intron, Korea) was used to extract genomic DNA from whole blood lymphocytes. *ADRA2A* polymorphisms were generated using oligonucleotide primers (5′-ACG TTG GAT GTT CTC CCA AGA TCC AGC TTC and 5′-ACG TTG GAT GCC TGC TGG GAG TTG GCC AT for the *ADRA2A* MspI [rs1800544] polymorphism; 5′-ACG TTG GAT GCT AAT TCC CCT TCC ATT CCC and 5′-ACG TTG GAT GGT GTA TAT TTA CAG CGG GG for the *ADRA2A* DraI [rs553668] polymorphism). The genotypes of the *ADRA2A* MspI variant were divided into C/C, G/C, and G/G; and those of the *ADRA2A* DraI variant were divided into C/C, C/T, and T/T. The detailed genotyping processes and the primer sequences used have been described previously [35].

### Statistical analysis

We compared the blood lead levels between ADHD and control groups using independent-sample t-tests and analysis of covariance (ANCOVA). In the ANCOVA, age, sex, and IQ, which were significantly different between groups, were used as covariates. A Pearson correlation analysis was used to examine the correlation of blood lead level with ADHD symptoms and neuropsychological functions in all participants. To analyze neuropsychological functions and ADHD symptoms on a spectrum, we analyzed the data from all participants concurrently. Pearson correlation analysis confirmed the neuropsychological functions and the symptoms of ADHD are associated with lead. Path analysis, which was performed to test the mediating effect of omission errors and response time variability, confirmed the correlation of the levels of lead and the effect on ADHD symptoms. Path model analysis was performed using the AMOS version 19.0 statistical program (SPSS Inc., USA). To evaluate the effect of the interaction between blood lead and genes on neuropsychological functions, hierarchical regression analyses were performed. The outcome variable was the CPT omission error; four genes (*DAT1, DRD4, ADRA2A* MspI, and *ADRA2A* DraI) were analyzed. For each regression, the age, sex, and IQ were entered in the first block; diagnosis (i.e., ADHD or control) was entered in the second block; genotypes and lead levels were entered in the third block; interaction terms between diagnosis or genotypes and lead levels (i.e., diagnosis × lead; *DAT1* VNTR × lead; *DRD4* VNTR × lead; *ADRA2A* MspI × lead; and *ADRA2A* DraI × lead) were entered in the fourth block; and interaction terms among diagnosis, genotypes, and lead levels (i.e., diagnosis × *DAT1* VNTR × lead; diagnosis × *DRD4* VNTR × lead; diagnosis × *ADRA2A* MspI × lead; and diagnosis × *ADRA2A* DraI × lead) were entered in the fifth block. Multiple linear regression analyses were used to examine the associations of blood lead levels with neuropsychological functions. Regression analyses were performed using a set of covariates that included age, sex, and IQ. To calculate the effect size, Cohen’s d, H or f2 was calculated. A Benjamini–Hochberg test with a false discovery rate threshold of 0.05 was performed to adjust for multiple comparisons for all analyses. All statistical analyses except the path analyses were performed using SPSS 22.0 (SPSS Inc., Chicago, IL, USA). Results were considered statistically significant at *p* < .05 (two-tailed).

## Results

### Demographic and clinical characteristics of participants

Table 1 shows the demographic and clinical characteristics, genotype frequencies, and environmental measures of 259 patients with ADHD and 96 controls. There were significant differences between the ADHD and control groups in mean age (8.8 vs. 10.5 years, *t* = 4.957, *F* = 11.306, *p* < .001, Cohen’s d = 0.63), male ratio (78.0 vs. 54.2%, χ^2^ = 19.531, *p* < .001, Cohen’s h = 0.51), and IQ (105.9 vs. 113.3, *t* = 4.957, *F* = 11.306, *p* < .001, Cohen’s d = 0.63). All subscales of the ADHD rating scale in the ADHD group were higher than were those in the control group (Inattention, 15.0 vs. 3.6, *t* = − 20.343, *F* = 15.542, *p* < .001, Cohen’s d = 2.76; hyperactivity-impulsivity, 10.2 vs. 1.7, *t* = − 17.769, *F* = 88.912, *p* < .001, Cohen’s d = 2.02; total, 25.2 vs. 5.5, *t* = − 21.239, *F* = 34.852, *p* < .001, Cohen’s d = 2.30). In CPT, omission errors (65.5 vs. 53.5, *t* = − 5.879, *F* = 33.913, *p* < .001, Cohen’s d = 0.70), commission errors (65.5 vs. 55.5, *t* = − 5.028, *F* = 16.181, *p* < .001, Cohen’s d = 0.58), and response time variability (63.3 vs. 50.8, *t* = − 6.402, *F* = 10.405, *p* < .001, Cohen’s d = 0.75) were higher in the ADHD group than in the control group. There was a significant difference in blood lead levels between the ADHD and control groups (1.4 vs. 1.3 μg/dL, *t* = − 2.818, *F* = 0.526, *p* = .005, Cohen’s d = 0.20). However, after adjusting for age, sex, and IQ, there was no significant difference in blood lead levels between the ADHD and control groups (*F* = 1.705, *R*^*2*^ = .070, *p* = .192). ANCOVA revealed that sex significantly affected differences in blood lead levels (*F* = 14.147, *R*^*2*^ = .070, *p* < .001). In analyses of males and females separately, males did not show a significant difference in blood lead levels between the ADHD and control groups (1.48 μg/dL vs. 1.42 μg/dL, *p* = .439). However, in females, average blood lead levels in the ADHD group were significantly higher than were those of the control group (1.31 μg/dL vs. 1.09 μg/dL, *p* = .011). In the whole group of patients and the control group, the lead concentration was higher in males than in females (1.5 vs. 1.2 μg/dL, t = 4.190, F = 1.187, *p* < .001, Cohen’s d = 0.54). No differences in any of the genotype frequencies were found between the ADHD and control groups.

**Table 1:** Demographic Characteristics of Participants

	Patients (*n* = 259)	Controls (*n* = 96)	*T or χ2*	*F*	*p* (Effect size)
Age, mean (*SD*) years	8.8 (2.4)	10.5 (3.0)	4.957	11.306	< .001^a^ (0.63*)
Male, *n* (%)	202 (78.0)	52 (54.2)	19.531		< .001^a^ (0.51**)
IQ, mean (*SD*)	105.9 (14.5)	113.3 (16.4)	4.100	1.668	< .001^a^ (0.48*)
ADHD subtypes, *n* (%)					
Combined	73 (28.4)				
Inattentive	98 (38.1)				
Hyperactive-impulsive	57 (22.2)				
Not otherwise specified	29 (11.3)				
ADHD Rating Scale, mean (*SD*)					
Inattention	15.0 (5.6)	3.6 (4.0)	−20.343	15.542	< .001^a^ (2.76*)
Hyperactivity-impulsivity	10.2 (6.5)	1.7 (2.0)	−17.769	88.912	< .001^a^ (2.02*)
Total	25.2 (10.7)	5.5 (5.7)	−21.239	34.852	< .001^a^ (2.30*)
Continuous Performance Test, mean (*SD*)					
Omission errors	65.5 (20.4)	53.5 (15.1)	−5.879	33.913	< .001^a^ (0.70*)
Commission errors	65.5 (19.2)	55.5 (15.2)	−5.028	16.181	< .001^a^ (0.58*)
Response time	55.4 (12.1)	54.4 (13.2)	−0.669	0.402	.504 (0.08*)
Response time variability	63.3 (17.9)	50.8 (15.4)	−6.402	10.405	< .001^a^ (0.75*)
Stroop Color-Word Test, mean (*SD*)					
Word reading score	44.0 (11.6)	49.0 (9.5)	0.692	3.755	< .001^a^ (0.47*)
Color naming score	45.4 (10.9)	50.9 (9.7)	0.846	4.244	< .001^a^ (0.53*)
Color-Word score	45.3 (12.5)	50.3 (11.9)	0.546	3.344	.001^a^ (0.41*)
Interference score	52.5 (11.3)	49.1 (13.8)	3.067	−2.311	.021^a^ (0.27*)
Environmental measure, mean (*SD*)					
Lead (μg/dL)					
Total	1.4 (.5)	1.3 (.5)	−2.818	0.526	.005^a^ (0.20*)
Male	1.5 (0.5)	1.4 (0.6)	0.775	0.808	.439 (0.18*)
Female	1.3 (0.5)	1.1 (0.3)	2.432	7.295	.017^a^ (0.49*)
Genotype					
*DAT1*, *n* (%)*			1.288		.525 (0.15**)
10/10	217 (83.8)	85 (88.5)			
With a 10	39 (15.1)	10 (10.4)			
Without 10	3 (1.2)	1 (1.0)			
*DRD4*, *n* (%)*			0.742		.690 (0.08**)
With 4/4	146 (56.4)	58 (60.4)			
With a 4	99 (38.2)	32 (33.3)			
Without 4	14 (5.4)	6 (6.3)			
*ADRA2A* MspI, *n* (%)*			0.470		.791 (0.03**)
C/C	30 (11.6)	12 (12.5)			
G/C	113 (43.6)	38 (39.6)			
G/G	116 (44.8)	46 (47.9)			
*ADRA2A* DraI, *n* (%)*			1.833		.400 (0.16**)
C/C	80 (30.9)	23 (24.0)			
C/T	125 (48.3)	53 (55.2)			
T/T	54 (20.8)	20 (20.8)			

*Note*: a: Items that are still significant after the multiple comparison adjustment with the 0.05 false discovery rate threshold which have a *p*-value <.05

*ADHD* attention-deficit/hyperactivity disorder, *IQ* intelligence quotient, *SD* standard deviation

*Cohen’s d values (0.80 = large, 0.50 = medium, 0.20 = small effect sizes)

**Cohen’s h values (0.80 = large, 0.50 = medium, 0.20 = small effect sizes)

### Correlation between lead and clinical characteristics

Table 2 shows the correlations between lead levels and ADHD symptoms/neuropsychological functions. The inattention and hyperactivity/impulsivity subscales of ADHD-RS, and omission errors and response time variability in CPT, showed positive correlations with blood lead levels. Age showed a negative correlation with blood lead levels. In the multivariable linear regression model, blood lead levels were associated with CPT omission errors (*B* = 3.748, 95% confidence interval 0.091–7.404, *p* = .045) and response time (*B* = 2.515, 95% confidence interval 0.013–5.017, *p* = .049), after adjusting for age, sex, and IQ. However, after adjustments for multiple comparisons, these associations were insignificant. (Table S1).

**Table 2:** Correlation between Lead and Clinical Characteristics (*n* = 355)

	*R*	*p*-value
Age	−.122	.022^a^
IQ	−.045	.403
Continuous Performance Test		
Omission errors	.158	.003^a^
Commission errors	.055	.304
Response time	.078	.151
Response time variability	.136	.010^a^
ADHD Rating Scale		
Inattention	.153	.004^a^
Hyperactivity-impulsivity	.181	.001^a^
Total	.173	.002^a^
Stroop Color-Word Test		
Word reading score	−.078	.157
Color naming score	−.057	.298
Color-Word score	−.013	.815
Interference score	.062	.272

*Note*: a: Items that are still significant after the multiple comparison adjustment with the 0.05 false discovery rate threshold which have a *p*-value <.05

*ADHD* attention-deficit/hyperactivity disorder, *IQ* intelligence quotient

### Mediating effects of neuropsychological functions on effects of lead on ADHD symptoms

**Fig. 1:**
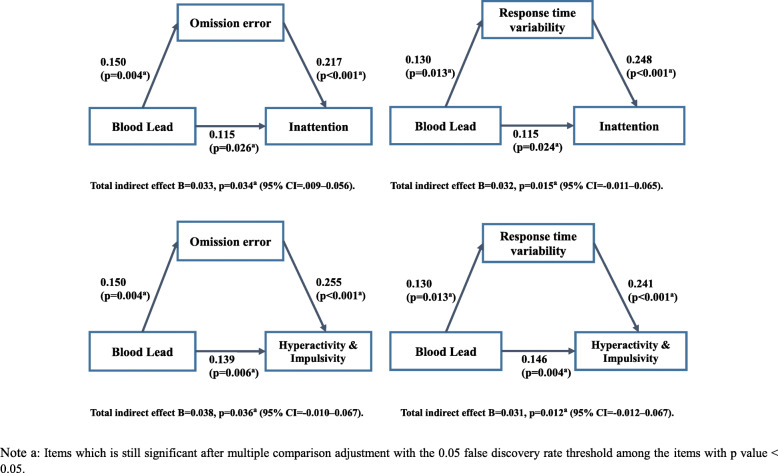
Cognitive control mediates blood lead effect on (ADHD) hyperactive-impulsive symptoms (*n* = 355, IQ covaried)

Omission errors and response time variability modified the effects of lead on ADHD symptoms after adjusting for IQ (Fig. 1). Both inattention and hyperactivity/impulsivity symptoms of ADHD were affected by lead, which was mediated by omission errors and response time variability in CPT, after adjusting for IQ.

### Association between genotypes, lead, and CPT scores

As shown in Table 3, no significant interactions were observed between *DRD4* VNTR and *ADRA2A* MspI genotypes, and blood lead levels in predicting CPT omission errors. Only the interaction between *ADRA2A* DraI genotype and blood lead level showed an association and medium effect size with CPT omission errors (*B* = 5.066, 95% confidence interval 0.197–9.934, *p* = .041). The most related genotype with omission errors was T/T, followed by C/T. The C/C genotype was found to be the least relevant. *DAT1* VNTR showed a trend for an association with omission error (*B* = 10.613, 95% confidence interval − 0.237–21.463, *p* = .055). However, after adjustments for multiple comparisons, these associations were insignificant. No interactions between lead and genes were associated with response time variability.

**Table 3:** Associations between Genotypes and Lead Interactions and CPT Scores (Omission Error and Response Time Variability) in the Multivariable Linear Regression Analysis

	Omission error^†^				Response time variability^†^			
Interaction	*B*	95% CI	*p*	f^2^	*B*	95% CI	*p*	f^2^
*DAT1* × Lead	10.613	−0.237, 21.463	.055	.274	−0.198	−10.527, 10.132	.970	.179
*DRD4* × Lead	−0.911	−7.380, 5.558	.782	.253	−4.065	−10.166, 2.036	.191	.188
*ADRA2A* MspI × Lead	2.870	−2.340, 8.079	.279	.262	−1.588	−6.526, 3.350	.527	.180
*ADRA2A* DraI × Lead	5.066	0.197, 9.934	.041	.269	3.392	−1.233, 8.017	.150	.186

*Note*: a: Items that are still significant after the multiple comparison adjustment with the 0.05 false discovery rate threshold which have a *p*-value <.05

*CI* confidence interval, *CPT* continuous performance test, *B* regression coefficient, f^2^ Cohen’s f^2^ values (.35 = large, .15 = medium, .02 = small effect sizes)

^**†**^ adjusted for intelligence quotient, age, and sex

## Discussion

This study compared blood lead levels in ADHD and control groups. We identified associations among blood lead levels, ADHD symptoms, and brain functions assessed by neuropsychological tests. We identified that a subset of genes, previously associated with ADHD, may impact brain functions through their interactions with lead. This study is the first to reveal that interactions between lead and particular genes affect brain functions mediating the effects of lead on ADHD symptoms.

Previous studies have reported associations of blood lead levels with ADHD symptoms or certain cognitive functions. Controversy remains regarding which domain of ADHD symptoms is more affected by lead. Research on patients with ADHD reported associations with hyperactivity-impulsivity [21], and a study conducted with a community sample revealed associations with impulsivity [34]. However, another study from a community sample reported that both inattention and hyperactivity were associated with blood lead levels [36], and a recent meta-analysis reported that effect sizes of the associations with inattention and hyperactivity-impulsivity were not substantially different [22]. This study confirmed that both of these symptoms were associated with blood lead levels. Since these two symptoms were strongly correlated when using Diagnostic and Statistical Manual of Mental Disorders (DSM) criteria, it may be difficult to test the hypothesis that blood lead levels have greater effects on either of the two symptoms [37]. Therefore, it is necessary to assess the effects of lead not only on symptoms identified in the diagnostic criteria, but also on measurable brain functions. The CPT is a neuropsychological test that has a strong correlation with clinical diagnoses and is considered a useful assessment tool for classifying patients [38]. In our study, omission errors and response time variability showed an association with lead; these two domains mediated the effects of lead on ADHD symptoms. There is controversy regarding the associations between lead levels and brain functions, as reports state that only commission errors (representing impulsivity) were positively associated with blood lead levels in a general population group [34], while other reports claim only a positive association for omission errors (reflecting inattention) [36]. In terms of mediator effect studies, one study hypothesized that blood lead would affect striatal-frontal circuitry and reported that response suppression and response time variability, related with this circuitry, partially mediated hyperactivity-impulsivity [21]. However, this study did not identify the mediation effects of the omission errors, which are known from a previous study to reflect only inattention [36]. Lead may damage dopaminergic pathways, which are associated with impulsivity, as well as cholinergic pathways, which are associated with attention [39]. Our study confirmed that omission errors were affected by blood lead levels, even after controlling for age, sex, and IQ. Further, omission errors was observed to mediate the effects of lead on both impulsivity and inattention. Therefore, our results suggest that the effects of lead on attention may affect both of these cardinal ADHD symptoms.

With regard to the etiology of ADHD, interactions between environment and genes have been reported [12, 40]. Lead is recognized as an environmental factor [3], and its potential to affect ADHD through interaction with genetic factors has been proposed [41]. Recent studies in a general population group reported effects of low lead levels on cognitive and executive functions [34, 36], emphasizing the need for a complex model that accounts not only for the direct effect of lead, but also other influences such as genetic susceptibility, for understanding the pathogenesis of ADHD. Our study confirmed that candidate ADHD genes, *ADRA2A* DraI and *DAT1* (trend level), affected omission errors by interacting with lead. The alpha-2A-adrenergic receptor is involved in executive functions in the prefrontal cortex, such as working memory, attention, and impulse control [42]. However, in a meta-analysis, the association between DraI polymorphisms and ADHD was not confirmed, as the odds ratio and *p*-value were .92 and .825, respectively [42]. To our knowledge, there have been no studies to date on the interactions between this gene and lead, although a recent study identified both the DraI polymorphism and blood lead level as factors predicting the therapeutic response to methylphenidate administration among patients with ADHD [15]. Results on the interactions between the DraI polymorphism and ADHD should be validated in future studies. Nevertheless, our study is the first to suggest that several genes are associated with the brain functions that mediate ADHD symptoms through their interactions with lead.

Previous studies have reported that lead has effects on cognitive functions and associations with intelligence. While no significant association with intelligence was observed in the present study, recent studies reporting lower average lead levels were able to demonstrate an association [21]. Future studies should analyze the potential modifying effect of other factors such as socioeconomic status (a covariate in other studies), or parents’ education levels.

In the comparison of blood lead levels in the ADHD and control groups, significantly higher blood lead levels were observed in the ADHD group. However, after correcting for age, sex, and IQ, there was no significant difference. Since the number of males in the ADHD group was greater than that of females, and the average blood lead levels were higher in males than in females overall, the average blood lead levels of the ADHD group were also higher than that of the control group. As previously reported, there was still a significant effect under 5 μg/dL, which is the standard level per Centers for Disease Control and Prevention. In addition, although males did not exhibit differences in blood lead levels between the ADHD and control groups, there was a significant effect on symptoms, suggesting that ADHD pathogenesis is also affected by interactions with other factors such as the genes identified in this study.

## Limitations and strengths

First, this study was unable to balance the proportion of males and females and match for age and sex between the ADHD and control groups. This limitation was addressed by using age and sex as covariates in the statistical comparisons between the ADHD and control groups. Because there are differences in genetic variation between races and countries, there are limitations in interpreting the data in this study. Since the distribution of the general population of Korea is not fully understood, the data of the patient population in this study needs to be compared with the genetic distribution of the general population and verified in the future. In addition, in diagnosing ADHD symptoms, the same symptoms are supposed to be identified in two different settings, but this study assessed symptoms from parents only. However, we increased the accuracy of diagnosis by using a structured assessment tool. Further, we did not classify participants by the presence of diagnosis in the analysis of associations between lead and symptoms. Instead, we considered a wide range of symptoms (from subclinical to clinical symptoms) for the statistical analyses. When considering multiple comparisons, several results were reported without statistical significance. However, the effect size was medium although it was not statistically significant in *p*-value. It is necessary to be careful in interpreting the results of this study. Since the statistical significance level is affected by the number of samples and variables, it is necessary to analyze with a larger number of samples in subsequent studies and check whether the same results are repeated. Lastly, the studies used as references in this paper did not include recent studies. This also means that research on the interaction of lead and gene expression has not been continued. Therefore, the results should be interpreted with caution. Subsequent studies on the effect of lead and gene interactions on the symptoms of ADHD should be continued. A strength of this study is that we attempted to understand the disorder across the spectrum based on changes in the DSM-5 and to calculate the effect size, Cohen’s d, H or f2 was calculated.

## Conclusions

Blood lead levels influenced ADHD symptoms, including both inattention and hyperactivity-impulsivity, and the effects were mediated by omission errors and response time variability, as measured by CPT. Omission errors were affected by interactions between lead and the *ADRA2A* DraI gene. It is necessary to confirm whether these results are replicable through additional studies on interactions of lead with relevant genes.

## Supplementary information

**Additional file 1: Table S1.** Association between Blood Lead Concentration and Clinical Characteristics in the ADHD Group in the Multivariable Linear Regression Analysis.

## Data Availability

The datasets analyzed during the current study are available from the corresponding author on reasonable request.

## Abbreviations

ADHD: attention deficit hyperactivity disorder
CPT: continuous performance test
DAT1: dopamine transporter
DRD4: dopamine D4 receptor
ADRA2A: alpha-2A adrenergic receptor gene
G-E: Gene–Environment interactions
SCWT: Stroop Color-Word Test
NMDA: N-methyl-d-aspartate
K-SADS-PL: Korean Kiddie Schedule for Affective Disorders and Schizophrenia–Present and Lifetime version
IQ: intelligence quotient
ADHD-RS: ADHD Rating Scale-IV
KEDI-WISC: Korean Educational Development Institute’s Wechsler Intelligence Scales for Children
ANCOVA: analysis of covariance
DSM: Diagnostic and Statistical Manual of Mental Disorders
