# Supplementary material for: Interaction between lead and noradrenergic genotypes affects neurocognitive functions in attention-deficit/hyperactivity disorder: a case control study
Source: BMC Psychiatry. 2020 Aug 6;20:407. doi: 10.1186/s12888-020-02799-3 (PMC7425170; doi:10.1186/s12888-020-02799-3)
Supplement: Supplementary file 1 — Additional file 1: Table S1. Association between Blood Lead Concentration and Clinical Characteristics in the ADHD Group in the Multivariable Linear Regression Analysis. [file 12888_2020_2799_MOESM1_ESM.docx]

Table S1

*Association^†^ Between Blood Lead Concentration and Clinical Characteristics in the ADHD Group in the Multivariable Linear Regression Analysis*

|  | *B* | 95% CI | *p* | *ƒ2* |
| --- | --- | --- | --- | --- |
| Continuous Performance Test |  |  |  |  |
| Omission errors | 3.748 | .091, 7.404 | .045 | .230 |
| Commission errors | -.925 | -4.412, 2.562 | .602 | .212 |
| Response time | 2.515 | .013, 5.017 | .049 | .028 |
| Response time variability | 2.647 | -.846, 6.140 | .137 | .129 |
| ADHD Rating Scale |  |  |  |  |
| Inattention | 1.053 | -.387, 2.493 | .151 | .134 |
| Hyperactivity-impulsivity | 1.259 | -.042, 2.560 | .058 | .191 |
| Total | 2.254 | -.278, 4.785 | .081 | .178 |
| Stroop Color-Word Test |  |  |  |  |
| Word reading score | -1.143 | -3.316, 1.031 | .302 | .164 |
| Color naming score | -.729 | -2.832, 1.375 | .496 | .155 |
| Color-Word score | .491 | -1.876, 2.857 | .684 | .213 |
| Interference score | 1.618 | -.963, 4.199 | .218 | .029 |

*Note*: a: Items which is still significant after multiple comparison adjustment with the 0.05 false discovery rate threshold among the items with p value < 0.05.

**^†^**: adjusted for intelligence quotient, age, and sex.

ADHD, attention-deficit/hyperactivity disorder; CI, confidence interval
